# Vitamin C as a Potential Geroprotector: Interactions with Aging Hallmarks and Implications for Healthy Longevity

**DOI:** 10.3390/nu18162704

**Published:** 2026-08-19

**Authors:** Diána Bodó, Ferenc Jójárt, Rebeka Jójárt, Dezső Csupor, Barbara Tóth

**Affiliations:** 1 Institute of Clinical Pharmacy, Faculty of Pharmacy, University of Szeged, H-6725 Szeged, Hungary; bodo.diana@szte.hu (D.B.); csupor.dezso@szte.hu (D.C.); 2 Goodwill Pharma Plc., H-6724 Szeged, Hungary; jojart.ferenc@goodwillpharma.com (F.J.); jojart.rebeka@goodwillpharma.com (R.J.); 3 Institute of Translational Medicine, University of Pécs, H-7622 Pécs, Hungary

**Keywords:** vitamin C, aging biomarkers, healthspan outcomes, antioxidant properties, biological functions

## Abstract

Vitamin C (L-ascorbic acid) is an essential micronutrient with established roles in antioxidant defense, epigenetic regulation, collagen synthesis, redox signaling, immune modulation, and cellular stress responses. This narrative review critically evaluates the potential role of vitamin C as a modulator of the hallmarks of aging. Although mechanistic studies suggest that vitamin C influences gene expression, inflammatory regulation, mitochondrial function, and cellular stress adaptation, the available evidence remains heterogeneous and is derived predominantly from in vitro and preclinical models, whereas human data are largely observational with limited interventional confirmation. Accordingly, vitamin C should be regarded as a context-dependent modulator of cellular resilience, with potential relevance under conditions of deficiency, inflammation, or increased oxidative stress rather than as a general anti-aging agent. Future studies integrating biomarkers of biological aging with clinically meaningful healthspan outcomes are needed to determine whether these mechanistic observations translate into measurable benefits during human aging.

## 1. Introduction

Population aging has emerged as one of the defining demographic and public health challenges of the 21st century. Advances in medicine, sanitation, nutrition and socioeconomic conditions have contributed to a remarkable increase in global life expectancy, resulting in an unprecedented expansion of the worldwide population of older adults. However, increases in lifespan have not necessarily been accompanied by proportional improvements in healthspan. Although individuals are living longer than ever before, many spend a substantial proportion of their later years affected by chronic diseases, frailty, impaired immune function, reduced physiological resilience and loss of functional independence. Aging is a multifactorial biological process associated with the development and increased susceptibility to chronic age-associated pathologies. More specifically, aging is the primary risk factor for most chronic diseases, including cardiovascular diseases, metabolic disorders, neurodegenerative conditions, cancers, and musculoskeletal decline, which all significantly contribute to morbidity, disability and healthcare utilization in later life. Consequently, the growing prevalence of multimorbidity among older individuals has become a major burden to healthcare systems, long-term care infrastructure, and socioeconomic resources globally [1,2,3].

The growing gap between healthspan and lifespan is a key concern in aging research, as a longer life does not necessarily imply better quality of life or a reduced burden of disease. Accordingly, the focus of aging research has shifted from lifespan extension alone toward the preservation of physiological function and postponement of age-associated pathologies [4].

These challenges have increased interest in prevention-oriented longevity medicine, which aims to target key biological mechanisms of aging before overt development of disease. Rather than addressing chronic diseases as separate conditions, this approach views aging as a modifiable process that contributes to multiple age-associated pathologies. By targeting the shared mechanisms of biological aging, longevity-focused interventions may offer the opportunity to delay multimorbidity and improve overall health trajectories at the population level. In this context, understanding these molecular and cellular drivers has become a central objective of aging research [5].

The biology of aging has undergone a profound conceptual shift over the past decade, moving from descriptive observations toward integrative, mechanism-based frameworks. Among these, the hallmarks of the aging framework have emerged as a central organizing paradigm, conceptualizing aging as the cumulative outcome of interconnected molecular and cellular processes, including genomic instability, epigenetic alterations, mitochondrial dysfunction and chronic inflammation [6]. This framework has been continually expanded, reflecting the growing recognition that aging is a dynamic, complex process rather than the simple linear decline of physiological functions. Certain biological processes associated with aging, such as low-grade inflammation or inflammaging, and the progressive decline of immune function, contribute to the development of multiple age-related pathologies across all organ systems [7]. Consequently, targeting fundamental mechanisms underlying the aging process has emerged as a unifying strategy to delay or prevent a broad spectrum of diseases. Within this context, regulatory systems that integrate metabolic and environmental signals have gained particular attention [8].

At the intervention level, longevity research has increasingly focused on both pharmacological and non-pharmacological modulators of pathways related to aging. Pharmacological geroprotectors currently being investigated include rapamycin, which is a mTOR inhibitor that modulates pathways associated with nutrient sensing and cellular growth, metformin, which influences insulin/AMPK signaling and systemic metabolic regulation, and senolytics, which selectively target and eliminate senescent cells contributing to chronic inflammation and NAD+ boosters, thereby restoring age-associated declines in cellular metabolic and mitochondrial function. Collectively, these compounds are thought to act on conserved biological processes underlying aging, thereby extending healthspan, as revealed by preclinical and early translational studies [9,10,11,12]. In parallel, nutritional and lifestyle-derived modulators of aging have received increasing attention. These modulators include spermidine, a natural polyamine associated with autophagy activation, polyphenols, which exert pleiotropic antioxidant and anti-inflammatory effects, and omega-3 fatty acids, which modulate lipid metabolism and inflammatory signaling and urolithin A, a gut microbiome-derived metabolite implicated in mitochondrial quality control via mitophagy. In addition, caloric restriction mimetics aim to reproduce the metabolic and stress-response benefits of dietary restriction without sustained energy limitation. Finally, the potential roles of selected vitamins and micronutrients are also being investigated to support cellular resilience, redox homeostasis, and immune function during aging [13,14,15,16].

While pharmacological geroprotection has shown promise, complementary approaches that are scalable and widely accessible remain of considerable interest [17,18,19,20]. As compared to pharmacological interventions, micronutrients and naturally occurring compounds may be advantageous in terms of accessibility, safety, and long-term translational applicability. The idea that small molecules and micronutrients can influence biological resilience is not new, as the pioneering work of Albert Szent-Györgyi on vitamin C anticipated the concept that specific biochemical factors may enhance systemic resistance to stress and disease, an idea closely aligned with current perspectives on healthy longevity [21]. Building on this foundation, increasing attention has been directed toward micronutrients and nutraceuticals as accessible modulators of aging-related pathways. Despite growing interest, the capacity of micronutrients (including vitamins) to meaningfully modulate core mechanisms underlying the aging process remains insufficiently resolved. The pleiotropic biological roles of vitamin C that extend beyond the classical function as an antioxidant have gained particular interest [22,23]. Vitamin C is actively transported and accumulated against a concentration gradient in several metabolically active tissues, including the adrenal glands, brain and central nervous system, pituitary gland, retina, and leukocytes, where intracellular concentrations may substantially exceed those in plasma. This tissue-specific distribution highlights the physiological importance of vitamin C beyond its role as a circulating antioxidant. Although the antioxidant properties are often emphasized, the biological functions of vitamin C also include well-established roles, such as acting as a cofactor in collagen biosynthesis, catecholamine production, carnitine synthesis, and peptide amidation. Moreover, vitamin C acts as a pleiotropic biological regulator involved in epigenetic remodeling, hypoxia signaling, redox homeostasis, inflammatory modulation, mitochondrial function and stem cell regulation, suggesting potential relevance across multiple hallmarks of aging [23,24,25,26]. Therefore, the aim of this review article is to critically evaluate whether the established biochemical functions of vitamin C provide a plausible mechanistic basis for involvement in aging biology and to summarize experimentally supported mechanisms and clinically confirmed effects related to healthy longevity. This narrative review summarizes experimental, translational, and clinical evidence identified through searches of PubMed and Web of Science using combinations of the terms “vitamin C”, “ascorbic acid”, “aging”, “hallmarks of aging”, “healthspan”, “longevity”, and related keywords. Priority was given to systematic reviews, meta-analyses, recent primary studies, and landmark papers relevant to the biology of aging. The literature search covered publications available from database inception through 2026. In addition to recent studies, landmark historical publications were considered, extending back to Albert Szent-Györgyi’s pioneering work on vitamin C in 1937, where relevant to the conceptual and mechanistic context of the review.

## 2. Systems-Level Biological Targets and Functional Domains of Vitamin C in Healthy Longevity

Although the putative anti-aging effects and the well-established antioxidant activity of vitamin C are intuitively linked, this relationship represents only one aspect of a far more complex biological role that extends well beyond direct scavenging of ROS. The role of vitamin C in biological aging cannot be adequately described within the traditional paradigm as an antioxidant. Instead, accumulating evidence supports vitamin C as a context-dependent regulatory molecule at the intersection of redox biology, epigenetic remodeling, immune regulation, and metabolic control [27]. The biological effects of vitamin C discussed in this section are derived from heterogeneous levels of evidence, ranging from mechanistic and cellular studies to preclinical in vivo models and limited translational or observational human data. Accordingly, these findings are discussed with appropriate caution, making a clear distinction between well-established mechanisms and effects that are supported mainly by preclinical studies, and broader system-level interpretations that should still be regarded as conceptual.

Currently available evidence suggests that vitamin C is unlikely to act as a classical geroprotective agent against a single hallmark of aging, as potential relevance to healthy aging is better understood in terms of involvement in several interconnected biological processes. This conceptual shift is essential to interpret the heterogeneous and sometimes contradictory experimental evidence.

### 2.1. Epigenetic Regulation: Vitamin C as a Cofactor of DNA Demethylation Systems

In aging and cellular senescence, vitamin C is generally considered to exert a potentially restorative effect to help preserve or re-establish epigenetic plasticity, partially reverse age-associated DNA hypermethylation patterns, and support more youthful transcriptional programs. Through these mechanisms, vitamin C may contribute to tissue homeostasis and regenerative capacity. As one of the most well-established mechanistic roles, vitamin C is an essential cofactor for Fe^2+^/2-oxoglutarate-dependent dioxygenases, particularly the TET family of enzymes (TET1/2/3), which catalyze the oxidation of 5-methylcytosine, thereby initiating demethylation of DNA. Vitamin C is therefore directly involved in the regulation of epigenetic plasticity, whereas the decline of this process with aging is associated with the loss of cellular identity, reduced regenerative capacity, and stem cell exhaustion [28]. Experimental studies, mainly with the use of cell cultures and animal models, have demonstrated that vitamin C can enhance TET enzyme activity, resulting in increased DNA demethylation, altered chromatin accessibility, improved somatic cell reprogramming efficiency, and stabilization of stem cell-like transcriptional states [29,30].

An important limitation of this evidence is that many of these observations have been obtained under non-physiological concentrations of vitamin C or with simplified in vitro systems. In addition, standard cell culture media (e.g., DMEM) do not contain vitamin C, and ascorbate undergoes rapid oxidation under standard culture conditions, making it difficult to maintain physiological concentrations unless regularly supplemented. These limitations raise questions regarding the magnitude and biological relevance of these effects in vivo, as the transport, compartmentalization, and turnover of vitamin C are tightly regulated. Consequently, the extent of the contribution of vitamin C-mediated TET activation to epigenetic remodeling at the organismal level remains incompletely understood, because current evidence supporting these long-term effects on human epigenetic aging trajectories has been primarily derived from observational associations between vitamin C intake or status and DNA methylation-based measures of aging, mechanistic evidence for TET-dependent epigenetic remodeling, and limited intervention data that are either genotype-specific or based on multinutrient formulations rather than vitamin C alone [31,32,33]. Furthermore, the role of vitamin C in epigenetic regulation appears to be strongly context-dependent, particularly when investigating the role of aging in cancer biology.

In contrast to aging, the same epigenetic mechanisms may have more complex and potentially dual effects in cancer biology, depending on the cell type, mutational background and tumor microenvironment [30]. In some cases, such as in TET2-mutant hematological malignancies, vitamin C may restore residual enzymatic activity and support more normal epigenetic regulation, potentially contributing to antitumor effects [34]. Also, increased epigenetic plasticity may promote tumor adaptation and heterogeneity. Thus, vitamin C-driven demethylation cannot be universally interpreted as beneficial or harmful in cancer [35,36], as the effects of vitamin C are dependent on the underlying genetic and metabolic states of the tumor. While preclinical studies support the anti- and pro-tumor effects of vitamin C depending on the context, clinical evidence remains limited and under active investigation. Although some observational studies have suggested inverse associations between circulating vitamin C concentrations and cancer risk [37], Mendelian randomization analyses have not supported a causal protective effect at physiological concentrations [38,39].

Although preclinical evidence strongly supports a mechanistically established role in DNA demethylation, the effects of vitamin C on long-term epigenetic and organismal aging trajectories in humans remain insufficiently demonstrated. Thus, the role of vitamin C in healthy aging should be considered a biologically plausible hypothesis but requires further validation with human studies.

### 2.2. Mitochondrial Dysfunction, Redox Signaling and Oxidative Resilience

Mitochondrial dysfunction is a central driver of biological aging and declining physiological resilience [40]. Excessive ROS production during mitochondrial dysfunction promotes damage to lipids, proteins and DNA, which drives genomic instability and cellular dysfunction, while overproduction of reactive nitrogen species intensifies redox imbalance in aging-associated inflammation. However, ROS also serve as signaling molecules that regulate mitochondrial biogenesis and adaptive stress responses [41]. At low or transient levels, ROS activate hormetic pathways, whereas chronic overproduction contributes to inflammaging and metabolic failure. Healthy aging therefore depends on the maintenance of redox homeostasis rather than the complete suppression of ROS production. Because mitochondria have limited capacity for DNA repair and are highly susceptible to oxidative damage, accumulated damage to mitochondrial DNA further impairs respiratory chain efficiency, reinforcing a self-amplifying cycle of mitochondrial dysfunction and ROS overproduction.

Vitamin C acts as a direct scavenger of ROS and nitrogen species, but its biological relevance extends beyond classical antioxidant activity to include the regulation of redox signaling, mitochondrial function, and systemic adaptation to stress [42]. Hence, vitamin C may support mitochondrial homeostasis via stabilization of mitochondrial membrane integrity, modulation of mitochondrial ROS production, preservation of nitric oxide bioavailability, and maintenance of endogenous antioxidant systems, while also participating in carnitine biosynthesis and indirectly facilitating mitochondrial fatty acid transport and energy metabolism. Through these interconnected mechanisms, vitamin C contributes to metabolic flexibility and energetic resilience, which both decline with aging. Beyond these metabolic roles, vitamin C is functionally linked to redox-sensitive cytoprotective signaling pathways [43], with emerging evidence highlighting interactions between vitamin C-dependent redox regulation and the Nrf2 antioxidant response system, a central regulator of cellular defense against oxidative and electrophilic stress. Nrf2 controls the expression of multiple detoxification and antioxidant enzymes involved in redox homeostasis and inflammatory regulation in aging tissues, suggesting that some of the biological effects of vitamin C are exerted by the modulation of stress regulation processes rather than direct antioxidant activity alone [44].

Several human studies support an association between vitamin C status and biomarkers of systemic oxidative stress, inflammation and endothelial dysfunction [45]. For example, plasma vitamin C concentrations are significantly lower in patients following ischemic stroke than in healthy controls, while circulating levels of CRP, ICAM-1, MCP-1 and F2-isoprostanes are elevated. Furthermore, vitamin C concentrations are inversely correlated with CRP and 8-epiPGF2α, suggesting that lower vitamin C status is associated with greater oxidative damage and inflammatory activation in vivo. These observations support the concept that vitamin C depletion may accompany states characterized by an increased oxidative–inflammatory burden and impaired vascular homeostasis [46,47,48]. Notably, several of these biomarkers are also characteristic features of aging and inflammaging, providing indirect evidence linking vitamin C status to biological processes associated with age-related decline in physiological resilience.

### 2.3. Vitamin C in the Context of Inflammaging and Chronic Inflammatory Activation

Inflammaging is a chronic, low-grade inflammatory state accompanying biological aging in the absence of infection. It arises from immune dysregulation, accumulation of senescent cells, impaired resolution of inflammatory responses, and persistent DAMP signaling, leading to sustained activation of innate immune pathways. Consequently a long-term pro-inflammatory milieu emerges that promotes endothelial dysfunction, tissue remodeling, metabolic decline, and an increased risk of age-related diseases, including cardiovascular and neurodegenerative disorders, with elevated levels of circulating markers, such as CRP, IL-6 and TNF-α [49]. Inflammaging is tightly coupled to oxidative stress, as chronic inflammation increases the production of ROS while simultaneously weakening endogenous antioxidant defenses, thereby establishing a self-reinforcing loop between redox imbalance and inflammatory signaling that drives progressive cellular and tissue dysfunction [50]. Although sepsis is an acute condition, it shares several pathophysiological mechanisms with inflammaging, including oxidative stress, immune dysregulation, endothelial dysfunction, and vitamin C depletion. In sepsis, these pathways become acutely dysregulated, leading to excess cytokine production, endothelial failure, oxidative injury, and depletion of endogenous antioxidants including vitamin C [51]. Vitamin C has been increasingly implicated in the regulation of these interconnected processes, extending beyond its classical antioxidant function to roles in endothelial protection, nitric oxide biology, and redox-sensitive immune signaling. Through these mechanisms, vitamin C may contribute to the maintenance of physiological homeostasis in both chronic and acute inflammatory states.

In severe systemic inflammatory conditions, particularly sepsis and septic shock, vitamin C has been investigated as a potential adjunctive therapy to modulate organ dysfunction, representing an acute clinical model of dysregulated inflammation and redox homeostasis relevant to aging biology [52]. Evidence from systematic reviews and meta-analyses has confirmed the benefits of intravenous vitamin C as an adjunctive therapy for patients with sepsis and septic shock, which are both characterized by profound oxidative stress, endothelial dysfunction, and dysregulated inflammatory activation. In a systematic review by Wang et al. (2023), which summarized clinical and translational evidence on vitamin C in sepsis-associated organ failure, vitamin C administration was associated with improvements in several physiological parameters related to vascular and organ function, although the findings were heterogeneous and did not consistently translate into improved survival outcomes [53]. Similarly, a meta-analysis by Cai et al. (2022) to evaluate randomized controlled trials of patients with septic shock receiving vitamin C in addition to standard care found that vitamin C treatment was associated with reduced vasopressor requirements and improved SOFA scores, suggesting potential benefits for circulatory stability and organ dysfunction, but did not consistently improve definitive clinical outcomes, including all-cause mortality ot intensive care unit length of stay [54]. Collectively, these findings suggest that vitamin C treatment may modulate physiological processes related to endothelial function, microcirculatory regulation and organ dysfunction in response to severe systemic inflammatory stress. Available clinical evidence supports the benefits of vitamin C against selected physiological and functional outcomes but does not demonstrate a clear mortality benefit. Nevertheless, these observations are consistent with the role of vitamin C in the regulation of redox balance and inflammatory responses under conditions of severe physiological stress.

### 2.4. Stem Cell Dysfunction in Aging and the Modulatory Role of Vitamin C

Vitamin C modulates the behavior of stem cells through multiple molecular pathways involved in cellular plasticity and fate regulation. A key pathway associated with vitamin C status involves modulation of hypoxia-inducible signaling through regulation of HIF-1α hydroxylase activity, thereby linking oxygen-sensing mechanisms to stem cell function. Experimental evidence indicates that vitamin C enhances transcription and stability of HIF-1α, as demonstrated in cellular models including renal carcinoma systems, highlighting a conserved biochemical mechanism of oxygen-dependent signaling modulation [54]. This oxygen-sensing axis is critical for maintaining appropriate cellular responses to hypoxic- or stress-related microenvironments, which is particularly relevant for stem cell maintenance and tissue regeneration. However, current evidence is derived primarily from cellular and experimental models; thus, the physiological significance of vitamin C in aging remains incompletely understood.

In addition, vitamin C has been implicated in the regulation of cellular reprogramming and stem cell state dynamics, suggesting a role in modulating the balance between self-renewal and differentiation [55]. These effects indicate that vitamin C may influence cellular plasticity and adaptive capacity in response to physiological stress. Within a broader conceptual framework, stem cell function is tightly linked to aging and malignant transformation processes, where dysregulation of cellular plasticity and stress adaptation contributes to both lower regenerative capacity and disease progression [56].

Collectively, available mechanistic evidence suggests that vitamin C may influence stem cell-related processes through multiple pathways. While these observations provide mechanistic support for the role of vitamin C in stem cell biology, direct human evidence demonstrating improvements in stem cell function or regenerative capacity during aging remains limited, warranting further validation with in vivo and human studies.

### 2.5. Cellular Senescence, Regenerative Decline, and the Potential Role of Vitamin C in Healthy Aging

As a key hallmark of biological aging that contributes to functional decline across tissues, cellular senescence is induced by diverse stressors, including genomic instability, epigenetic changes, mitochondrial dysfunction, telomere attrition, oncogene activation and chronic inflammatory stress, all converging via persistent signaling by DNA damage response pathways [57]. Cellular senescence also activates ATM/ATR-CHK pathways and leads to p53-mediated arrest of the cell cycle, accompanied by increased expression of p21 and p16, apoptosis resistance, metabolic reprogramming, and chromatin remodeling (including the formation of SAHF) as well as SA-β-gal activity [58]. A defining feature of senescent cells is the SASP, a proinflammatory program involving cytokines (e.g., IL-6, IL-8), chemokines, growth factors, and matrix-remodeling enzymes that disrupt tissue homeostasis and promote chronic inflammation. SASP regulation is mediated by several pathways, such as NF-κB, C/EBPβ, p38 MAPK, mTOR and cGAS-STING, linking DNA damage sensing to inflammatory and innate immune signaling [11]. Senescent cells actively drive tissue dysfunction rather than serving as passive markers of aging, as even in low numbers they impair stem cell niches, reduce regenerative capacity, and contribute to the onset of cardiovascular, metabolic, fibrotic, and neurodegenerative diseases [59]. Circulating SASP-related factors, such as GDF15, STC1, and MMP1, have been proposed as biomarkers of the burden of senescence, while experimental clearance of p16INK4a- or p21CIP1-positive cells has been shown to improve tissue function and extend healthspan in preclinical models [60].

Current evidence does not support classification of vitamin C as a senolytic agent. Instead, vitamin C is more appropriately viewed as a factor that may influence biological processes associated with cellular senescence. Through its role as a cofactor for Fe^2+^/2-oxoglutarate-dependent dioxygenases, including TET DNA demethylases and JmC-domain histone demethylases, vitamin C contributes to the epigenetic regulation of chromatin accessibility, transcriptional stability and cellular identity [61]. These mechanisms are relevant to senescence because age-associated epigenetic drift contributes to the loss of cellular function, stem cell exhaustion, and impaired regenerative capacity. Rather than directly eliminating senescent cells, vitamin C influences molecular pathways involved in cellular responses to stress and regulation of gene expression. However, direct evidence demonstrating that vitamin C prevents cellular senescence, reduces the burden of senescent cells, or improves senescence-related outcomes in humans remains limited. Overall, the proposed relationship between vitamin C and cellular senescence is supported primarily by mechanistic evidence that indirectly links epigenetic regulation, oxidative stress, and inflammatory signaling. While these observations are promising, potential effects on the burden of senescent cells and regenerative decline remain insufficiently established in human studies [62].

### 2.6. Telomere Maintenance in Aging: The Role of Oxidative Stress and Vitamin C-Dependent Redox Modulation

Telomere length is dynamically regulated by the balance between replicative attrition and maintenance mechanisms, reflecting intrinsic cellular processes and extrinsic environmental stressors. Progressive shortening of telomeres occurs partly due to the replication of the telomere ends, which leads to incomplete replication of chromosomal termini during successive cell divisions [63]. In addition, although providing a compensatory elongation mechanism, telomerase activity is limited in most somatic tissues and is therefore insufficient to fully prevent progressive loss of the telomere ends. As a result, critically shortened telomeres activate DNA damage response pathways, leading to permanent cell cycle arrest and the establishment of cellular senescence [64]. These processes link telomere dynamics to the progressive functional decline characteristic of biological aging. However, telomere shortening is widely considered not only a potential contributor to aging but also a biomarker reflecting the biological aging process. Accordingly, direct evidence demonstrating that modification of telomere length alters human aging trajectories remains limited.

This interpretation is further supported by integrative evidence indicating that telomere shortening during aging may be attenuated by interventions with antioxidant and anti-inflammatory agents, highlighting the interconnected roles of oxidative stress and inflammatory processes in the regulation of telomere dynamics and biological aging [65]. Antioxidant defense systems may indirectly influence telomere dynamics by mitigating oxidative damage to DNA. In this regard, vitamin C, as a major water-soluble antioxidant, has been proposed to contribute to the preservation of telomere integrity by reducing oxidative stress-mediated DNA damage. Observational studies of human populations have reported positive associations between higher circulating or dietary vitamin C status, and telomere length of leukocytes, suggesting that improved antioxidant micronutrient status may be linked to telomere preservation [66]. Similarly, longitudinal data suggest that higher micronutrient intake may be associated with slower telomere attrition over time, although the strength and consistency of these associations varied across nutrients and study designs. Furthermore, experimental and population-based evidence indicates that antioxidant supplementation may mitigate oxidative stress-mediated telomere shortening, providing a plausible mechanistic link between vitamin C-related antioxidant capacity and telomere maintenance [66,67]. However, current evidence remains largely observational and fails to establish a causal relationship between vitamin C intake and telomere preservation. Moreover, there is a lack of direct evidence that vitamin C supplementation can slow telomere attrition or improve aging-related outcomes through telomere-dependent mechanisms.

### 2.7. Autophagy as a Redox-Sensitive Stress Response Pathway in Aging: Modulation by Vitamin C

Autophagy is a lysosome-dependent degradation pathway that is essential for cellular quality control, proteostasis, and metabolic adaptation under physiological and stress conditions [67]. The regulation of autophagy is tightly integrated with nutrient- and energy-sensing pathways, as well as redox signaling networks, positioning autophagy as a central node in cellular stress responses and aging-related homeostatic control. ROS play an important role in regulating autophagy. Beyond the potential to cause damage, ROS can also act as signals that shape autophagic flux. Under physiological conditions, moderate increases in ROS production can activate autophagy as an adaptive survival mechanism by promoting the removal of damaged proteins and organelles, whereas excessive or chronic ROS accumulation may dysregulate autophagic processes and contribute to cellular dysfunction and pathological outcomes [68]. Most evidence supporting the role of ROS in autophagy regulation originates from cellular and animal models, whereas direct evidence in humans remains limited.

Vitamin C influences autophagic pathways in a biological-context-specific manner. Experimental evidence indicates that ascorbate can induce autophagy under certain conditions, potentially enhancing cellular stress adaptation and survival through improved degradation of damaged cellular components. However, predominantly in cancer models, ascorbate-induced autophagy may also be associated with pro-death signaling in specific cellular contexts, suggesting that this effect is not unidirectional but rather strongly dependent on the cell type, redox environment, and stress intensity [68]. Whether this context-dependent mechanism also contributes to healthy aging and longevity remains to be determined through future experimental and clinical studies. Accordingly, vitamin C cannot be regarded as a universal activator or inhibitor of autophagy. Rather, available evidence suggests that vitamin C may influence autophagic responses under specific biological conditions. Experimental evidence for vitamin C-mediated modulation of autophagy is predominantly derived from in vitro and preclinical studies, with context-dependent effects observed across different cellular models [69]. Therefore, the role of vitamin C in the regulation of autophagy should be considered biologically plausible but has not yet been established in clinical and aging-related contexts.

### 2.8. Interactions Between Vitamin C, Redox Signaling, and Nutrient-Sensing Pathways

Nutrient-sensing pathways are increasingly recognized as central regulators of aging biology by integrating cellular responses to energy availability, metabolic stress, and environmental signals. Among the most important nutrient-sensing systems are AMPK, mTOR, and the sirtuin family of NAD+-dependent deacetylases collectively regulate cellular metabolism, autophagy, mitochondrial function, stress responses and stem cell maintenance [69]. Dysregulation of these pathways has been associated with reduced metabolic flexibility, chronic inflammation, impaired regeneration and loss of physiological resilience during aging. AMPK acts as a cellular energy sensor that is activated under low-energy conditions and promotes catabolic processes, such as fatty acid oxidation, glucose uptake, and autophagy, while inhibiting anabolic signaling [70]. In contrast, chronic dysregulation of mTOR signaling has been linked to impaired autophagy, metabolic imbalance, and increased cellular senescence, whereas sirtuins integrate nutrient availability with mitochondrial function, epigenetic regulation, and stress-response pathways through NAD+-dependent mechanisms, thereby linking the metabolic state to longevity-associated cellular programs [71].

Nutrient-sensing pathways are tightly linked to intracellular redox homeostasis, thereby forming an integrated regulatory network that coordinates metabolic adaptation and stress responses during aging. Oxidative stress and mitochondrial dysfunction modulate signaling by AMPK, mTOR and sirtuins. However, direct evidence demonstrating that vitamin C regulates these signaling pathways in aging-relevant in vivo contexts remains limited. Age-associated disruption of nutrient sensing has been associated with impaired tissue regeneration and stem cell dysfunction. Within this interconnected regulatory environment, vitamin C may indirectly influence nutrient-sensing networks by contributing to intracellular redox balance. Accordingly, vitamin C should be more appropriately viewed as a factor that may influence the redox environment under which these pathways operate rather than as a direct regulator of nutrient-sensing signaling [72]. Moreover, vitamin C may influence biological processes associated with these nutrient-sensing pathways through effects on cellular redox balance, although direct evidence for clinically relevant modulation by vitamin C during human aging remains insufficient.

### 2.9. Gut Microbiome, Host–Microbial Interactions, and the Potential Role of Vitamin C in Healthy Longevity

The gut microbiome is increasingly recognized as a key regulator of healthy aging. Age-associated shifts in microbial composition have been linked to chronic low-grade inflammation, metabolic dysfunction, immune dysregulation and impaired intestinal barrier integrity. These changes typically involve reduced microbial diversity, loss of beneficial commensals, and expansion of pro-inflammatory pathobionts, leading to disruption of host–microbiome homeostasis [73]. Increased intestinal permeability and translocation of microbial-derived molecules further promote persistent innate immune activation, which contributes to inflammaging and progressive functional decline [74]. Beyond its role in digestion, the gut microbiome functions as a metabolic and immunological interface that influences nutrient sensing, mitochondrial activity, redox balance, and immune regulation [75]. Microbial metabolites, including SCFAs, bile acid derivatives, and tryptophan metabolites, modulate pathways involved in inflammation control, epithelial integrity, and metabolic adaptation [76]. Accordingly, gut dysbiosis may contribute to reduced metabolic flexibility and diminished regenerative capacity in aging tissues.

Diet is one of the strongest modulators of gut microbial composition and function, and increasing evidence suggests that dietary antioxidants may influence host–microbiome interactions through redox-sensitive mechanisms [77]. Vitamin C indirectly contributes to the maintenance of intestinal and systemic homeostasis through modulation of oxidative balance within the gastrointestinal environment [78]. Experimental and emerging translational evidence suggests that vitamin C status influences the microbial composition of the gut, intestinal barrier integrity, and redox-sensitive inflammatory pathways involved in oxidative mucosal injury [79]. Accumulating evidence further indicates that oxidative stress within the intestinal microenvironment contributes to dysbiosis by altering microbial survival conditions and promoting inflammatory microbial signatures. In this context, vitamin C can be viewed as one component of dietary antioxidant exposure that may contribute to the maintenance of redox-sensitive host–microbiome interactions through modulation of oxidative and inflammatory stress conditions rather than acting as a microbiome-specific regulatory agent [80]. However, direct evidence demonstrating clinically meaningful modulation of the aging-associated microbiome by vitamin C remains limited.

### 2.10. Role of Vitamin C in Gap Junctional Intercellular Communication

Intercellular communication is a central feature of aging biology that links molecular and cellular damage to progressive tissue dysfunction and organismal decline. Among the main mechanisms of intercellular communication, gap junction-mediated communication allows cells to coordinate functional responses and may therefore play an important role in how aging processes unfold at the tissue level. Gap junctions enable the direct exchange of small signaling molecules, including Ca^2+^, IP_3_, and cAMP, thereby synchronizing cellular activity within tissues and maintaining functional coherence across cellular networks. As a result, gap junctional signaling is increasingly recognized as a critical component of tissue homeostasis, while dysregulation contributes to age-associated functional decline [81,82]. Gap junctional communication is highly sensitive to redox imbalance and regulated through signaling pathways responsive to oxidative stress. ROS influence the phosphorylation status of connexins and channel permeability via kinase-dependent mechanisms, thereby linking cellular redox status to the regulation of intercellular coupling. A potential role of vitamin C based on antioxidant properties has been proposed in the regulation of gap junctional intercellular communication, although direct experimental validation in the context of aging is currently unavailable [83,84,85,86].

## 3. Life-Stage and Condition-Dependent Modulation of Vitamin C Requirements

Across different stages of life and physiological conditions, vitamin C status should be interpreted as a dynamic variable influenced by metabolic demand, oxidative stress burden, and inflammatory activation. Although these factors may increase vitamin C utilization and have led to the hypothesis that some groups, particularly older adults, could benefit from higher vitamin C intake, current evidence remains insufficient to conclude that absolute vitamin C requirements are consistently increased in response to these conditions. Nevertheless, certain physiological and pathological conditions, including obesity, diabetes, acute infections, heat stress, and intense physical exercise, may reduce vitamin C status despite intake at the RDA, and higher intakes may therefore be required to maintain adequate plasma and tissue concentrations. Therefore, the available data support consideration of condition-specific differences in vitamin C status but not a universal increase in recommended intake.

### 3.1. Older Adults

Older adults are considered a population at an increased risk of inadequate vitamin C status due to reduced dietary intake, multimorbidity, chronic inflammation, polypharmacy, functional decline and institutionalization [87]. However, current evidence suggests that healthy aging itself is not consistently associated with lower vitamin C status or substantially increased physiological requirements for vitamin C. Comparative studies have generally found no major differences in circulating vitamin C concentrations and pharmacokinetic responses between healthy younger and older adults, indicating that aging per se does not appear to significantly impair the absorption, distribution, or overall handling of vitamins [88]. In contrast, lower vitamin C concentrations are more frequently observed in frail, hospitalized and institutionalized older individuals, where inadequate dietary intake, chronic disease burden, and increased metabolic demands may contribute to vitamin C insufficiency or deficiency. Evidence suggests that institutionalized individuals may require higher vitamin C intake to achieve adequate circulating concentrations, although this finding appears to reflect health status rather than chronological age [89].

Current dietary recommendations for vitamin C are not increased for older adults. The RDA remains at 90 mg/day for men and 75 mg/day for women, whereas the tolerable UL for adults is 2000 mg/day. Available evidence does not indicate that healthy aging necessitates modification of these recommendations or the established safety limits for vitamin C intake [90]. From a clinical perspective, maintaining adequate vitamin C status in later life may be particularly relevant because older adults are more vulnerable to malnutrition and micronutrient deficiencies. In support of this concept, a large population-based MRI study of 2044 community-dwelling older adults demonstrated that higher plasma vitamin C concentrations were associated with greater gray and white matter volumes and more favorable structural connectivity of the DMN, particularly in the posterior cingulate cortex. Moreover, DMN connectivity was positively associated with cognitive performance, suggesting that adequate vitamin C status may contribute to preserving brain structural integrity and cognitive function during aging [88]. Preclinical evidence suggests that vitamin C deficiency can contribute to skeletal muscle atrophy and impaired physical performance, supporting the importance of adequate vitamin C status for maintaining physical function during aging [90].

These findings do not indicate increased physiological requirements of vitamin C for healthy older adults, but rather highlight the potential consequences of deficiency in vulnerable populations. Overall, the goal is to maintain an adequate, continuous vitamin C status tailored to different life stages. In older age, the key factor is not necessarily extremely high doses but a stable and sustained supply of vitamin C, which reflects the balance among intake, absorption, metabolism, and excretion [89,90,91]. Certain compromised or adverse life situations may be associated with a temporarily increased requirement for vitamin C intake. In fact, the available literature suggests that the primary concern in the older population is not age-related alterations to vitamin C pharmacokinetics or safety, but rather the increased prevalence of factors that predispose to inadequate vitamin C status.

### 3.2. Exercise and Stress

Lifestyle- and exposure-related factors substantially influence vitamin C homeostasis by modulating the burden of oxidative stress, inflammatory signaling, and antioxidant turnover. Physical exercise, particularly when intense or prolonged, is associated with increased production of ROS, which leads to transient oxidative stress and elevated antioxidant demand. In this context, the potential of vitamin C to attenuate exercise-induced oxidative damage and support recovery has been extensively investigated [92,93]. Regular exercise alone has not been consistently shown to increase vitamin C requirements; however, in athletes and individuals exposed to prolonged physical strain, particularly under heat stress, intake greater than the minimum recommended levels—approximately 200–250 mg/day—has been proposed to support adequate vitamin C status [94,95].

Psychological and physiological stressors similarly influence vitamin C homeostasis through activation of the HPA axis and sympathetic nervous system, resulting in increased secretion of cortisol and catecholamines, along with enhanced ROS production. This stress-induced redox imbalance may increase systemic antioxidant demand and accelerate vitamin C turnover. Vitamin C has been proposed to play an active role in the coordinated stress response rather than functioning solely as a dietary antioxidant. Vitamin C acts as an essential component of an evolutionarily conserved “stress hormone” system, functioning in parallel with classical stress mediators. Stress exposure is associated with rapid mobilization and depletion of vitamin C, particularly from adrenal stores, where it is present at high concentrations and released during activation of the HPA axis [96]. Available evidence from systematic reviews and mechanistic studies indicates that no evidence-based dosage recommendation can be established for vitamin C supplementation in the context of exercise-induced stress or physiological stress responses.

### 3.3. Acute and Chronic Illnesses

Acute critical illnesses, such as sepsis and septic shock, are characterized by dysregulation of the host response to infection, which leads to systemic inflammation, endothelial injury, and multiorgan dysfunction. Vitamin C status is consistently reduced, reflecting increased metabolic turnover, redistribution, and consumption under conditions of severe oxidative and inflammatory stress [97]. Clinical and translational evidence suggests that vitamin C may serve as a supportive adjunct for the management of sepsis, although robust large-scale clinical trials are still needed to clarify the therapeutic efficacy, optimal dosing strategies, safety profile and appropriate patient selection [98]. Mechanistically, studies of immune cell function further indicate altered compartmental handling of vitamin C during sepsis, including preserved intracellular concentrations in neutrophils despite systemic depletion, suggesting active cellular uptake and prioritization under conditions of deficiency. Overall, vitamin C dynamics in acute illnesses reflect a state of heightened metabolic demand and non-homeostatic redistribution rather than stable physiological regulation. In clinical studies of septic shock, intravenous vitamin C has been administered at doses of 25 mg/kg every 6 h; however, current evidence remains insufficient to establish an optimal therapeutic regimen, thus further large-scale randomized trials are required [99]. In contrast, chronic disease states are associated with long-term alterations to endothelial function, metabolic signaling, and redox homeostasis rather than acute depletion. Vitamin C has been implicated in vascular and metabolic regulation via oxidative stress modulation and endothelial protection. In diabetic complications, including diabetic retinopathy, experimental and translational evidence suggests that vitamin C may contribute to vascular protection and modulation of oxidative injury, potentially influencing disease progression through redox-sensitive pathways. Similarly, epidemiological evidence from dietary pattern analyses indicates that higher intake of plant-derived nutrients, including nitrate-rich foods, is associated with favorable long-term blood pressure trajectories, as vitamin C may participate indirectly through modulation of oxidative stress and bioavailability of nitric oxide. Unlike acute clinical illnesses, chronic conditions do not typically involve overt depletion of circulating vitamin C but rather reflect subtle, sustained perturbations in redox balance and vascular homeostasis, where vitamin C may contribute to long-term functional maintenance rather than acute physiological rescue. According to Lykkesfeldt et al., the effects of vitamin C in chronic disease contexts are primarily related to dose–response relationships within physiological ranges rather than disease-specific pharmacological requirements. Plasma saturation of vitamin C is typically achieved at intakes of approximately 100-200 mg/day, with most functional and vascular effects reaching a plateau around this range. A prior review suggests that the benefits observed in cardiovascular and metabolic health are largely associated with intakes above the deficiency-preventing level of 85-110 mg/day, whereas higher intakes do not appear to provide additional clinically relevant advantages. Overall, in chronic diseases, vitamin C is considered a contributor to long-term redox and vascular homeostasis rather than a high-dose therapeutic agent [100,101].

## 4. Conclusions and Future Perspectives

Taken together, the mechanisms discussed across the hallmarks of aging suggest that vitamin C may influence multiple biological processes associated with aging, including epigenetic regulation, mitochondrial function, inflammatory responses, stem cell biology, autophagy, nutrient sensing, telomere maintenance, gut physiology, and intercellular communication. These processes are highly interconnected, as alterations to one pathway may influence the functions of others. Within this context, vitamin C appears to act primarily through effects on redox balance and related cellular processes rather than a single aging-specific mechanism. Importantly, current evidence is heterogeneous and derived predominantly from mechanistic and preclinical studies, with comparatively limited validation in aging human populations (Table A1). For transparency, the characteristics of the studies discussed in Section 2 are summarized in Supplementary Table S1. Accordingly, the framework presented in this review should be interpreted as an integrative biological model supported by varying levels of evidence across individual hallmarks of aging rather than a validated causal model of vitamin C-mediated longevity.

The contemporary geroscience paradigm views aging as a dynamic network of interconnected biological processes rather than a collection of independent pathological events. Within this framework, vitamin C emerges as a biologically plausible modulator of multiple hallmarks of aging, including epigenetic regulation, mitochondrial function, redox homeostasis, inflammaging, stem cell maintenance, cellular senescence, autophagy, nutrient sensing, and tissue-level communication [102].

As a central conclusion of this review, the effects of vitamin C should not be interpreted solely through the traditional antioxidant paradigm. Instead, accumulating evidence supports a broader role of vitamin C in coordinating redox-sensitive, metabolic, and stress-response pathways that collectively influence cellular resilience and physiological adaptability during the aging process. The biological significance of vitamin C appears to arise less from direct radical scavenging and more from its capacity to modulate the regulatory environment in which aging-associated processes operate.

The current body of evidence positions vitamin C as a promising candidate within the emerging field of geroscience. Currently, much of the mechanistic rationale originates from experimental and preclinical studies, while clinical and translational investigations providing direct evidence demonstrating effects on biological aging trajectories, healthspan extension or longevity outcomes in humans remain limited. The effects of vitamin C appear highly context-dependent, varying according to dose, tissue-specific metabolism, nutritional status, disease burden, and physiological stress conditions. Recent analyses of vitamin C biology during the aging process have similarly emphasized that its influence on longevity-related outcomes is likely mediated through complex interactions among metabolic, immune, and redox-regulatory networks rather than a singular mechanism of action [103].

**Figure A1:**
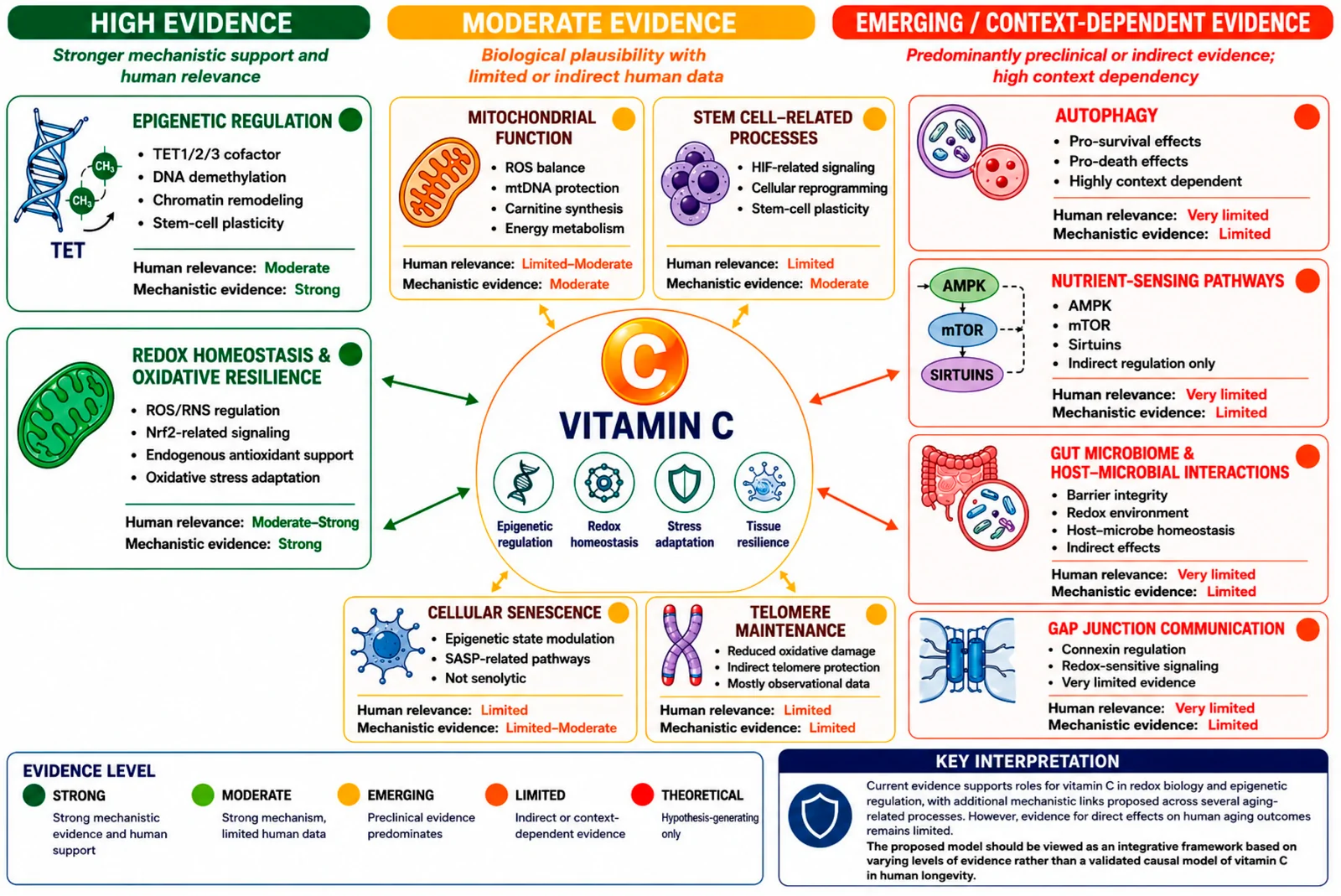
Vitamin C and biological aging: strength of evidence.

Nevertheless, vitamin C fulfills several conceptual criteria as a potential geroprotective factor. Contemporary geroscience increasingly evaluates candidate geroprotectors according to the ability to simultaneously influence multiple interconnected hallmarks of aging and to improve organismal resilience across age-related biological systems. In this regard, vitamin C exhibits a remarkably broad spectrum of biological activities, intersecting with epigenetic remodeling, mitochondrial maintenance, inflammatory regulation, and adaptive stress responses (Figure A1). Such pleiotropic actions distinguish vitamin C from interventions targeting a single pathway and position vitamin C within a system-level framework of healthy longevity. As the understanding of aging biology continues to evolve, vitamin C represents an intriguing example of a nutrient with diverse biological functions that align with several key principles of contemporary longevity research [104].

Future research should move beyond antioxidant models and focus on integrated investigations combining mechanistic biology, biomarkers of biological age, longitudinal human cohorts, and rigorously designed clinical interventional studies. Particular attention should be directed toward the identification of context-specific conditions under which vitamin C-dependent pathways may influence biological aging, including interactions with nutritional status, multimorbidity, frailty, and age-associated immune dysfunction. Current evidence is also insufficient to define the optimal plasma vitamin C concentrations or supplementation dosages required to achieve potential geroprotective effects in healthy humans, highlighting the need for dose-finding clinical studies. Future studies should further investigate the interactions of vitamin C with other geroprotective interventions, including pharmacological agents (rapamycin and metformin) and dietary strategies, to determine their potential synergistic or antagonistic effects on healthy aging. Within the emerging concepts of precision geromedicine, vitamin C intake may ultimately prove most valuable not as a simple standalone longevity intervention but as a component of broader resilience-promoting strategies targeting the interconnected biology of aging.

In conclusion, vitamin C appears to be a promising system-level candidate geroprotector, with biological effects that intersect with several hallmarks of aging and processes relevant to longevity. These diverse biological activities provide a strong rationale for continued investigations into the potential contributions of vitamin C to sustained health and longevity. The key translational challenge is to determine whether vitamin C status or supplementation can measurably influence biological aging trajectories, healthspan-related outcomes, or validated biomarkers of aging.

## Data Availability

No new data were created or analyzed in this study. Data sharing is not applicable to this article.

## Supplementary Materials

The following supporting information can be downloaded at: https://www.mdpi.com/article/10.3390/nu18162704/s1, Table S1: Summary of study design, sample size, key conclusions, and evidence level for references [29,30,31,32,33,34,35,36,37,38,39,40,41,42,43,44,45,46,47,48,49,50,51,52,53,54,55,56,57,58,59,60,61,62,63,64,65,66,67,68,69,70,71,72,73,74,75,76,77,78,79,80,81,82,83,84,85,86].

## Abbreviations

The following abbreviations are used in this manuscript:

8-epiPGF2α	8-epi-prostaglandin-F2α
AMPK	Activated protein kinase
ATM kinase	Ataxia telangiectasia mutated kinase
ATP	Adenosine triphosphate
ATR kinase	Ataxia telangiectasia and Rad3-related kinase
BCL-2	B-cell lymphoma 2
cAMP	Cyclic adenosine monophosphate
C/EBPβ	CCAAT/enhancer-binding protein β
cGA-STING	Cyclic GMP-AMP synthase- stimulator of interferon genes
CHK1	Checkpoint kinase 1
CHK2	Checkpoint kinase 2
CRP	C-reactive protein
DAMPs	Danger-associated molecular patterns
DMN	Default mode network
DNA	Deoxyribonucleic acid
DDR	DNA damage response
GDF15	Growth differentiation factor 15
HIF-1α	Hypoxia-inducible factor-1 α
HPA	Hypothalamic-pituitary-adrenal
ICAM-1	Intercellular adhesion molecule 1
IL-6	Interleukin-6
IL-8	Interleukin-8
IP_3_	Inositol 1,4,5-triphosphate
JmC domain	Jumonji C domain
MCP-1	Monocyte chemoattractant protein-1
MMPs	Matrix metalloproteinases
MMP1	Matrix metalloproteinase 1
mTOR	Mechanistic target of rapamycin
NAD+	Nicotinamide adenine dinucleotide
NF-κB	Nuclear factor κ B
Nrf2	Nuclear factor erythroid 2-related factor 2
p16INK4a	Tumor suppressor protein, cyclin-dependent kinase inhibitor 2A
p21CIP1	Regulatory protein, cyclin-dependent kinase inhibitor 1A
p38 MAPK	p38 mitogen-activated protein kinase
RDA	Recommended Dietary Allowance
SA-β-gal	Senescence-associated β-galactosidase
SAHF	Senescence-associated heterochromatin foci
SASP	Senescence-Associated Secretory Phenotype
SCFA	Short-chain fatty acid
SOFA	Sequential Organ Failure Assessment
STC1	Stanniocalcin 1
TET	Ten-Eleven Translocation
TNF-α	Tumor necrosis factors-α
UL	Upper intake level

## Appendix A

**Table A1:** System-level biological targets of vitamin C in healthy longevity.

Hallmark/Biological Domain	Description and Impact on Aging	Proposed Role of Vitamin C
**2.1. Epigenetic regulation**	Age-associated epigenetic drift contributes to loss of cellular identity, impaired regeneration and stem cell exhaustion through altered DNA methylation and chromatin accessibility.	Vitamin C acts as a cofactor for TET dioxygenases and histone demethylases, potentially supporting epigenetic plasticity and transcriptional stability.
**2.2. Mitochondrial dysfunction and oxidative resilience**	Mitochondrial impairment promotes ROS accumulation, metabolic dysfunction, inflammaging and reduced energetic resilience during aging.	Vitamin C modulates mitochondrial redox balance, supports endogenous antioxidant systems and influences oxidative stress adaptation.
**2.3. Inflammaging and chronic inflammatory activation**	Persistent low-grade inflammation contributes to endothelial dysfunction, tissue remodeling, frailty and age-related disease progression.	Vitamin C supports inflammatory homeostasis through redox-sensitive immune and endothelial regulatory pathways.
**2.4. Stem cell dysfunction**	Aging-associated stem cell exhaustion reduces tissue regeneration and adaptive cellular plasticity.	Vitamin C influences stem cell maintenance, reprogramming capacity and hypoxia-related signaling pathways.
**2.5. Cellular senescence**	Accumulation of senescent cells and SASP signaling contributes to chronic inflammation, tissue dysfunction and regenerative decline.	Vitamin C influences senescence-associated epigenetic and metabolic states.
**2.6. Telomere attrition**	Progressive telomere shortening promotes genomic instability, DNA damage signaling and cellular senescence.	Antioxidant effects of vitamin C reduce oxidative telomeric DNA damage and support telomere maintenance indirectly.
**2.7. Autophagy dysregulation**	Impaired autophagic clearance contributes to proteostatic failure, accumulation of damaged organelles and metabolic imbalance.	Vitamin C may modulate autophagy through redox-sensitive signaling pathways, with both pro-survival and pro-death effects reported.
**2.8. Nutrient-sensing dysregulation**	Altered AMPK, mTOR and sirtuin signaling impairs metabolic adaptation, stress responses and tissue regeneration during aging.	Vitamin C may influence nutrient-sensing pathways through modulation of intracellular redox balance and metabolic stress responses.
**2.9. Gut microbiome dysbiosis**	Age-related microbial alterations contribute to inflammation, impaired barrier integrity and metabolic dysfunction.	Vitamin C may support host–microbial homeostasis through antioxidant and redox-sensitive mechanisms within the intestinal environment.
**2.10. Intercellular communication dysfunction**	Impaired gap junctional signaling disrupts tissue coordination, stress adaptation and cellular homeostasis during aging.	Vitamin C may support redox-sensitive signaling processes involved in maintenance of intercellular communication.
