# Supplementary material for: Vitamin C as a Potential Geroprotector: Interactions with Aging Hallmarks and Implications for Healthy Longevity
Source: Nutrients. 2026 Aug 19;18(16):2704. doi: 10.3390/nu18162704 (PMC13517069; doi:10.3390/nu18162704)
Supplement: Supplementary file 1 [file nutrients-18-02704-s001.zip › nutrients-4471478-supplementary.pdf]

Table S1: Summary of study design, sample size, key conclusions, and evidence level for references [29–86].

Reference	Review section	Study design	Sample size	Verified conservative conclusion	Evidence level
[29] A. Zhitkovich et al., 2020	Epigenetic regulation	Narrative review	Not applicable	Summarizes vitamin C-dependent epigenetic regulation, particularly its cofactor role for TET and JmjC dioxygenases and its potential effects on DNA/histone demethylation.	Mechanistic review
[30] B. Pereira et al., 2024	Epigenetic regulation	Narrative review	Not applicable	Describes nuclear and cytoplasmic functions of vitamin C, including redox regulation and cofactor-dependent control of epigenetic enzymes.	Mechanistic review
[31] J. Guz et al., 2017	Epigenetic regulation	Narrative review	Not applicable	Reviews epigenetic reprogramming as a potential strategy to reverse age-associated epigenetic changes; evidence remains predominantly preclinical.	Mechanistic review
[32] A. Keshawarz et al., 2023	Epigenetic regulation	Epigenome-wide association meta-	11,866 paricipants across eight population-	Identifies associations between dietary/nutrition	Human observational

		analysis	based cohorts	al exposures, including vitamin C-related measures, and DNA methylation patterns.	
[33] S. Li et al., 2026	Epigenetic regulation	Large-scale randomized controlled trial	COSMOS included 2,815 samples from 985 participants	Reports effects of a multinutrient intervention on DNA methylation-based aging measures; the independent contribution of vitamin C cannot be isolated.	Human interventional; multinutrient
[34] L. Bordoni et al., 2024	Epigenetic regulation	Observational study	The study analyzed data from the LifeLines Cohort, a large prospective cohort and Biobank. LifeLines is a multidisciplinary prospective population-based cohort study which examines 167,729 persons living in the North of the Netherlands, 760 individuals from the LifeLines-DEEP Cohort	Reports associations between diet quality/EpiNutrient intake and epigenetic age; findings are associative and vulnerable to residual confounding.	Human observational

[35] K. Schönberger et al., 2017	Epigenetic regulation	Commentary / perspective	Not applicable	Highlights preclinical evidence that vitamin C may enhance residual TET activity and promote differentiation-related antileukemic effects in selected genetic contexts.	Preclinical interpretation
[36] K. Linowiecka et al., 2020	Epigenetic regulation	Narrative review	Not applicable	Reviews how vitamin C transport and intracellular availability may have context-dependent roles in carcinogenesis and tumor biology.	Mechanistic review
[37] L. Cimmino et al., 2018	Epigenetic regulation	Narrative review	Not applicable	Concludes that vitamin C can modulate epigenetic reprogramming and cancer biology, with effects depending on cell type, genotype, dose, and metabolic context.	Mechanistic/preclinical review
[38] D. Aune et al., 2018	Epigenetic regulation	Systematic review and meta-analysis	69 cohort studies included, prsopetive design	Higher dietary or circulating antioxidant levels, including vitamin C, were associated with lower risks of cardiovascular disease, cancer, and mortality.	Human observational
[39] H. Chen et al., 2022	Epigenetic regulation	Two-sample Mendelian	The SNPs associated with vitamin C	Found no convincing evidence that genetically	Human genetic causal inference

		randomization	were selected from a genome-wide association study (GWAS) meta-analysis of vitamin C consisting of 52,018 individuals	predicted vitamin C status causally associated with overall cancer risk.	
[40] Y. Fu et al., 2021	Epigenetic regulation	Two-sample, bi-directional Mendelian randomization	Genome-wide association study (GWAS) meta-analysis on 52,018 Europeans	Did not support a causal protective effect of circulating vitamin C concentration on the five most common cancers in Europe populations	Human genetic causal inference
[41] Y. Guo et al., 2023	Mitochondrial/redox signaling	Narrative review	Not applicable	Reviews mitochondrial dysfunction as a central component of aging, emphasizing impaired bioenergetics, altered dynamics, mtDNA damage, and redox imbalance.	Mechanistic review
[42] I. Liguori et al., 2018	Mitochondrial/redox signaling	Narrative review	Not applicable	Summarizes the bidirectional relationship between oxidative stress, aging, and age-related diseases; excessive oxidative damage contributes to functional	Mechanistic review

				decline.	
[43] T. Kietzmann, 2023	Mitochondrial /redox signaling	Narrative review	Not applicable	Reviews vitamin C roles beyond antioxidant scavenging, including oxygen sensing, dioxygenase activity, and epigenetic regulation.	Mechanistic review
[44] Pant et al., 2024	Mitochondrial /redox signaling	Narrative review	Not applicable	Reviews Nrf2 activation as a regulator of redox-sensitive immune networks and a potential therapeutic target in inflammatory disease.	Mechanistic review
[46] S. Sasikumar et al., 2023	Mitochondrial /redox signaling	Preclinical study (Cd-exposed H9c2 cells from rat cardio myoblast cell line)	Not stated in manuscript	Ascorbic acid attenuated cadmium-associated cardiac hypertrophy and injury, with effects linked to activation of Nrf2-related antioxidant signaling.	Preclinical
[47] X.Tang et al., 2024	Mitochondrial /redox signaling	Systematic review / meta-analysis	Multiple studies	Higher vitamin C intake was associated with lower ischemic-stroke risk, but heterogeneity and observational designs limit causal interpretation.	Human evidence synthesis
[48] C. Sánchez-Moreno et al.,	Mitochondrial /redox signaling	Case-control observational	15 ischemic stroke patients and 24	Stroke patients had lower plasma vitamin C and higher	Human observational

2004		study	controls	inflammatory/oxidative-stress markers than controls, supporting an association with acute oxidative-inflammatory burden.	
[49] A. W.Ashor et al., 2015	Mitochondrial/redox signaling	Systematic review and meta-analysis of randomized controlled trials	46 randomized controlled trials (1817 participants)	Antioxidant supplementation modestly improved endothelial function, with effects varying by baseline health and intervention characteristics.	Human interventional synthesis
[50] I. M. Rea et al., 2018	Inflammaging	Narrative review	Not applicable	Reviews chronic inflammatory triggers and cytokine pathways as contributors to age-related disease and functional decline.	Mechanistic review
[51] B. J. Andonian et al., 2025	Inflammaging	Narrative review	Not applicable	Proposes an integrated inflammaging framework linking chronic low-grade inflammation with multiple aging-related diseases.	Mechanistic review
[52] J. Joffre et al., 2021	Inflammaging	Narrative review	Not applicable	Reviews oxidative stress and endothelial dysfunction as key mechanisms of vascular and organ injury in sepsis and acute inflammation.	Mechanistic/clinical review

[53] Z. Wang et al., 2023	Inflammaging	Narrative review	Not applicable	Concludes that vitamin C may improve selected physiological or organ-function parameters in sepsis, but survival benefits remain inconsistent.	Mechanistic/clinical review
[54] B. Cai et al., 2022	Inflammaging	Systematic review and meta-analysis	13 studies comprised 6 cohort studies and 7 randomized controlled trials	Vitamin C was associated with improved SOFA scores and reduced vasopressor requirements, but not with consistent reductions in mortality or ICU length of stay.	Human interventional synthesis
[55] C. Wohlrab et al., 2019	Stem-cell biology	In vitro experimental study on ccRCC cell lines	Not stated in manuscript	Increasing intracellular ascorbate enhanced HIF-hydroxylase activity and altered hypoxia-pathway signaling, demonstrating a cellular oxygen-sensing mechanism.	Preclinical
[56] J. Fu et al., 2020	Stem-cell biology	Narrative review	Not applicable	Reviews vitamin C as a modulator of stem-cell state and reprogramming in cancer-related contexts; the manuscript citation metadata appear inconsistent and should be checked.	Mechanistic review
[57] D. J. Rossi et	Stem-cell biology	Narrative review	Not applicable	Reviews shared stem-cell	Mechanistic review

al., 2008				pathways in tissue aging and malignant transformation, emphasizing declining regenerative capacity and dysregulated self-renewal.	
[58] A. Ajoolabady et al., 2025	Cellular senescence	Narrative review	Not applicable	Summarizes defining hallmarks, mechanisms, and regulation of cellular senescence and its contribution to aging and disease.	Mechanistic review
[11] S. Chaib et al., 2022	Cellular senescence	Narrative review	Not applicable	Reviews senescent-cell biology and the translational development of senolytic approaches, while noting unresolved efficacy and safety issues.	Mechanistic review
[59] Y. Sun et al., 2023	Cellular senescence	Narrative review	Not applicable	Emphasizes that senescent cells are heterogeneous across triggers, tissues, and states, complicating biomarker selection and therapeutic targeting.	Mechanistic review
[60] D. McHugh et al., 2025	Cellular senescence	Narrative review	Not applicable	Reviews cellular senescence as a therapeutic target in cancer and age-related disorders, balancing	Mechanistic review

				detrimental SASP effects against context-dependent protective roles.	
[61] R. Di Micco et al., 2021	Cellular senescence	Narrative review	Not applicable	Concludes that senescent cells actively contribute to tissue dysfunction and that targeting them can improve outcomes in preclinical models, although human translation is still limited.	Mechanistic review
[62] J. P. Brabson et al., 2021	Cellular senescence	Narrative review	Not applicable	Reviews vitamin C-dependent epigenetic mechanisms that support genomic stability, particularly through TET and JmjC dioxygenases.	Mechanistic review
[63] S. Reichert et al., 2017	Telomeres	Narrative review	Not applicable	Finds that oxidative stress is generally associated with telomere shortening in vivo, while methodological heterogeneity limits firm quantitative conclusions.	Preclinical review
[64] K. N. Prasad et al., 2017	Telomeres	Narrative review	Not applicable	Reviews telomere shortening as a contributor to cellular senescence and aging-related dysfunction.	Mechanistic review

[65] A. Sen et al., 2014	Telomeres	Cross-sectional observational study	785 individuals	Higher plasma lutein, zeaxanthin, and vitamin C concentrations were associated with longer leukocyte telomere length.	Human observational
[66] J-Y. Lee et al., 2017	Telomeres	Population-based cohort study	1985 Koreans	Earlier consumption of vitamin C, folate and potassium can delay biological ageing in middle-aged and older adult	Human observational
[67] M. A. Levy et al., 2024	Telomeres	Experimental study (cultures of neonatal human fibroblast cells)	Not stated in manuscript	A multivitamin mixture reduced oxidative-stress-mediated telomere shortening in the experimental model; the effect cannot be attributed specifically to vitamin C.	Preclinical review
[68] R. Scherz-Shouval et al., 2011	Autophagy	Narrative review	Not applicable	Reviews ROS as both signaling mediators and damaging agents in autophagy regulation, with outcomes depending on intensity, duration, and cellular context.	Mechanistic review
[69] L. Sedlackova et al., 2020	Autophagy	Narrative review	Not applicable	Describes reciprocal interactions among NAD metabolism, ROS, and autophagy in cellular health and aging.	Mechanistic review
[70] J. L.	Autophagy	Experime	Not stated	Reports context-	Preclinical

Welsh et al., 2012		ntal study (pancreatic cancer cells)	in manuscript	dependent ascorbate-induced autophagy, which may contribute either to cell death or survival; full methodological detail is limited in the abstract.	review
[71] G. R. Steinberg et al., 2023	Nutrient sensing	Narrative review	Not applicable	Reviews current mechanisms of AMPK activation and its roles in metabolic adaptation, mitochondrial homeostasis, and autophagy.	Mechanistic review
[72] R. A. Saxton et al., 2017	Nutrient sensing	Narrative review	Not applicable	Reviews mTOR as a central integrator of nutrient, growth-factor, and energy signals controlling growth, metabolism, and autophagy.	Mechanistic review
[73] R. H. Houtkooper et al., 2012	Nutrient sensing	Narrative review	Not applicable	Reviews sirtuins as NAD+-dependent regulators of metabolism, stress responses, and healthspan.	Mechanistic review
[74] Y. R. Li, 2024	Nutrient sensing	Narrative review	Not applicable	Summarizes vitamin C functions in redox biology and clinical contexts; it does not provide direct evidence that vitamin C regulates nutrient-sensing pathways in human aging.	Mechanistic review

[75] N. V. Kanimozhi et al., 2025	Gut microbiome	Narrative review	Not applicable	Reviews age-associated microbiome changes and potential therapeutic approaches, emphasizing dysbiosis, barrier dysfunction, and inflammation.	Mechanistic review
[76] A. Ticinesi et al., 2026	Gut microbiome	Narrative review	Not applicable	Reviews mechanisms linking the gut microbiome with aging trajectories and interventions against ageing (personalized, microbiome-centred)	Mechanistic review
[77] M. S. R. Rajoka et al., 2021	Gut microbiome	Narrative review	Not applicable	Reviews bidirectional interactions between food antioxidants and the gut microbiome, including effects on microbial composition and host redox/inflammatory status.	Mechanistic review
[78] N. Zmora et al., 2019	Gut microbiome	Narrative review	Not applicable	Concludes that diet is a major determinant of gut-microbiota structure and function, with downstream metabolic and immune effects.	Mechanistic review
[79] A. T. Otten et al., 2021	Gut microbiome	Pilot intervention study	14 participants	Vitamin C supplementation shifted gut bacterial community composition and	Human interventional

				increased selected short-chain-fatty-acid-related taxa; small sample size limits generalizability.	
[80] H. Vergauwen et al., 2015	Gut microbiome	Preclinical study (IPEC-J2 cells)	Not applicable	Trolox and ascorbic acid reduced direct and indirect oxidative stress and supported epithelial-barrier-related outcomes in the experimental model.	Preclinical review
[81] H. Yan et al., 2015	Gut microbiome	Preclincial experimental study	n=18 C57BL/6J male mice weighing 22-25 g were obtained	Ascorbic acid reduced oxidative stress and inflammatory injury in experimental colitis.	Preclinical review
[82] N. Naliyadhara et al., 2023	Gut microbiome	Narrative review	Not applicable	Reviews how dietary antioxidants may modulate gut microbiota and host inflammatory/redox pathways; direct aging-specific clinical evidence remains limited.	Mechanistic review
[83] H. Sun et al., 2025	Intercellular communication	Narrative review	Not applicable	Reviews exosomes as mediators of intercellular communication relevant to tissue homeostasis and healthy aging.	Mechanistic review
[85] A. Yamada et al.,	Intercellular communication	Preclincial experime	Dental epithelial cell	Demonstrates a role for connexin 43-mediated	Preclinical review

2021		ntal study	cultures from Krt14-RFP mice	gap-junction communication in tissue development; it does not directly test vitamin C or aging.	
[86] B. L. Upham et al., 2009	Intercellular communication	Narrative review	Not applicable	Proposes that gap junction-mediated redox signaling is a critical regulator of gene expression and tissue homeostasis in oxidative stress-related diseases.	Mechanistic review
